# Identifying Critically Ill Patients at Risk of Malnutrition and Underfeeding: A Prospective Study at an Academic Hospital

**DOI:** 10.15171/apb.2019.037

**Published:** 2019-06-01

**Authors:** Fatemeh Osooli, Saeed Abbas, Shadi Farsaei, Payman Adibi

**Affiliations:** ^1^Department of Clinical Pharmacy and Pharmacy Practice, Isfahan University of Medical Sciences, Isfahan, Iran.; ^2^Anesthesiology and Critical Care Research Center, Isfahan University of Medical Sciences, Isfahan, Iran.; ^3^Department of Clinical Pharmacy and Pharmacy Practice, Isfahan Pharmaceutical Sciences Research Center, Isfahan University of Medical Sciences, Isfahan, Iran.; ^4^Department of Gastroenterology, Integrative Functional Gastroenterology Research Center, Isfahan University of Medical Sciences, Isfahan, Iran.

**Keywords:** Food intake, Malnutrition, Intensive care unit, Nutritional decline

## Abstract

***Purpose:*** Malnutrition is highly prevalent in critically ill patients and is associated with the increased healthcare-related cost and poor patient outcomes. Identifying the factors associated with undernutrition may assist nutritional care. Therefore, this study was designed to identify factors associated with malnutrition and inadequate energy intake to improve nutritional support in intensive care unit (ICU).

***Methods:*** This prospective study was conducted on 285 random samples of ICU patients. We reported time to initiate the enteral nutrition, percent of the adequately received nutrition, and development of malnutrition during the follow-up period. Moreover, variables and clinical outcomes associated with calories underfeeding and malnutrition were reported.

***Results:*** In 28.6% of samples, enteral feeding was initiated greater than 48 hours after ICU admission. During follow-up, 87.4% and 83.3% of patients failed to receive at least 80% of protein and energy target, and malnutrition developed in 84% of study population. Moreover, surgical and medical patients compared to trauma patients were associated with underfeeding. However, only nutrition risk in the critically ill score (NUTRIC) score ≥5 could predict malnutrition development in our study. Finally, underfeeding contributed significantly to a more mortality rate both in ICU and hospital.

***Conclusion:*** Our findings revealed that the majority of nutritionally high-risk patients failed to receive adequate calories and subsequently developed malnutrition. The present study added valuable information to the small body of literature about the factors affecting nutritional decline and malnutrition during the ICU stay.

## Introduction

An appropriate nutritional support is indispensable to critically ill patients, who are almost at the hyper-metabolic state of their clinical condition such as trauma, sepsis, and major surgery.^1^ These critical conditions result in a disproportional release of cytokine and stress hormones that alter energy and protein metabolism and eventually lead to malnourishment.^2^

A recent systematic review revealed the strikingly high prevalence of malnutrition in intensive care unit (ICU) patients (ranged from 38% to 78%), which is associated with the patients’ increased morbidity, mortality, and hospital-related cost.^3^ The increased dependency on mechanical ventilation, length of hospital stay, ICU readmission, rate of infection, and risk of hospital mortality associated with undernutrition, make it an important dilemma in the care of ICU patients.^4,5^

It should be considered that not all critically ill patients are at risk of developing adverse events related to nutritional issue.^6^ The nutrition risk in the critically ill score (NUTRIC score) has been recently proposed in order to identify patients who will benefit from an aggressive nutritional support.^6^ In other words, NUTRIC score was considered to determine which critically ill patient is at high nutrition risk.^7^

There are variety of scores and tools to identify patients’ nutritional status, including evaluation of nutrition-related factors, nutritional intake, and energy expenditure.^8^ In the context of nutrition-related factors, the body mass index (BMI), physical examination, anthropometric data, and some biochemical indicators such as serum level of albumin and prealbumin were routinely used to monitor nutritional status.^9,10^ However, low levels of these biochemical factors revealed both nutritional condition and persistent physiological stress related to the underlying illness.^11,12^

To evaluate the adequacy of nutritional intake, the percentage of required energy which was received by the patients, was applied in different studies.^8,13^ Although different formulas have also been developed to estimate energy requirement, the indirect calorimetry is currently advocated for measuring energy demands in critically ill patients.^14^

Various protocols and guidelines were published to give information about distinct aspects of clinical nutrition. However, the guideline of nutrition support therapy in adult ICU patient of Society of Critical Care Medicine and American Society for Parenteral and Enteral Nutrition was mostly used in clinical studies to determine the nutritional status of patients.^15^ Although, little is known regarding risk factors of undernutrition in ICU patients until now.^16^ Therefore, our ultimate goal is to investigate the prevalence of undernutrition among ICU patients, determine certain conditions related to malnutrition and underfeeding of them, and eventually evaluate their possible relationship with the mortality rate and duration of the ICU and hospital stay.

## Materials and Methods

A prospective analytical study was conducted in 285 random samples of tertiary referral academic ICUs at Al-Zahra hospital in Isfahan in 2017. These wards with more than 60 beds are the largest ICUs in the middle of Iran that admit patients from a primary or secondary health professional. Adult patients (at least 18 years of age) were recruited within 24 hours of ICU admission and followed up 7 to 10 days to evaluate the study hypothesis. The patients who were expected to stay less than 7 days did not include in this study, and those received any oral nutrition support before 7 days were excluded. Moreover, patients who met study endpoint defined as oral diet, death, or discharge from ICU before 7 days were excluded.

Patients were prospectively evaluated, and the related data were gathered. Histories of nutrition intake and weight loss before ICU admission were collected during the interview with patients or their family members or the review of patients’ charts. Patients’ charts were used to extract data on demographics and admission category (medical, surgical, or trauma). Data variables required to calculate Acute Physiology and Chronic Health Evaluation (APACHE II), Sequential Organ Failure Assessment (SOFA), and NUTRIC score (age, APACHE II, SOFA, comorbidities, and days in the hospital prior to ICU admission) were also collected at baseline (on ICU admission).

Nutritional data, which included the amounts of enteral or parenteral nutrition prescribed and actually received, were also recorded daily, up to the study endpoint or to a maximum 10 days. Any discrepancies between these amounts were evaluated, and their reasons were described. These data were also used to calculate macronutrients (energy, protein, carbohydrates, and lipids) pertaining to nutrition prescriptions and intakes. Manufacturers’ information was used to calculate the amount of each macronutrient in the enteral nutrition formula (hospital or commercial).

Adequacy of calories and protein intake from nutrition therapy was considered as 80%-120% of the goal-feeding amounts. This way, the number of calories or grams of received protein were divided by the estimated daily requirements. Less than 80% of calories’ intake was reflected as underfeeding, while over-feeding occurred when administered calories meet more than 120% of patients’ caloric requirement. In the similar manner, the percentage of 80%-120% was defined as rational, while less than 80% or more than 120% was considered irrational intake.

The goal of the caloric requirement was determined by Harris-Benedict equation by taking into account of stress and activity factors.^17^ Recommendations of a physician, pharmacotherapist, and dietitian were also deliberated to determine goal-feeding regimen. We also considered the required daily intake of carbohydrates and lipids accounting for 60%-70% and 30%-40% of caloric intake, respectively, based on patients’ clinical condition. In addition, the goal for protein intake was 1.2-2 g/kg, depending on comorbidities and severity of clinical diseases.^15,18,19^

To measure the albumin and prealbumin, blood samples were obtained from the recruited patients ICU admission and on the 10th day of the follow-up. Although the role of albumin and prealbumin as the biomarkers to determine malnutrition was limited due to false positive and negative results but, various studies nowadays showed prealbumin <15 mg/dL could be associated with malnutrition.^9,10^

The type of nutrition (enteral or parenteral), time to initiate enteral or parenteral nutrition from ICU admission, and prevalence of iatrogenic underfeeding were also reported. Finally, the outcomes of patients were recorded as the length of stay and mortality rate both in ICU and hospital.

### Statistical analysis

Data were analyzed using SPSS software version 19.0 (SPSS Inc., Chicago, IL, USA). Descriptive statistics (mean and standard deviation) were performed to characterize the demographic data including APACHE II score, prescribed and received calories and proteins, time to initiate enteral nutrition, and length of ICU stay. Categorical variables including NUTRIC score, type of nutrition, prevalence of iatrogenic underfeeding and malnutrition based on prealbumin level and mortality rate, were reported as count and percentage.

The received and optimal amounts of calories and proteins were compared with one sample t-test. In addition, logistic regression was performed to evaluate the association of demographic and baseline clinical factors with the prevalence of optimal feeding and malnutrition. Firstly, a univariate analysis was run including all characteristics. Thereafter, a multivariable model with selected variables (with *P* < 0.05 in univariate analysis or considered risk factors) was constructed to calculate odds ratios (OR) and its confidence intervals. Finally, more analyses were done to compare qualitative and quantitative data in independent groups by chi-square and ANOVA test, respectively. A *P* value less than 0.05 was considered significant in all analyses.

## Results and Discussion

**Figure 1:**
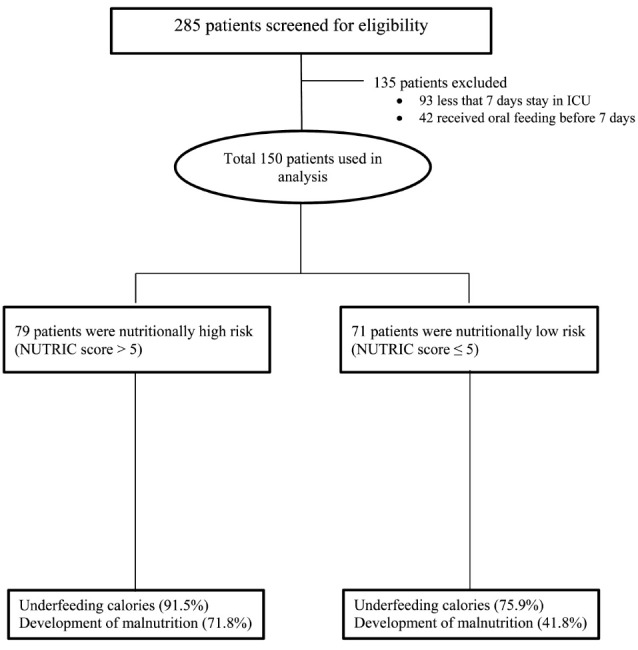
Flow chart of patients’ enrolment, and follow-up in subgroups of nutritional risk. NUTRIC: nutrition risk assessment in critically ill.

Among 285 enrolled patients, 135 patients were excluded because they stayed less than 7 days in ICU (n = 93) or received oral feeding (n = 42). Therefore, 150 patients completed the study to be included for more analysis (Figure 1). The mean age was 57.42 ± 17.20 years old with 58% male. Moreover, 79 patients (52.7%) were identified as high nutritional risk (based on NUTRIC score ≥5), 53 patients (35.3%) were with a prealbumin <15 mg/dL and 127 (84.7%) with a BMI <25. More demographic and baseline clinical characteristics were provided in Table 1.

**Table 1:** Demographic and clinical baseline characteristics of participants upon admission

**Variables**	**Total (N = 150)**
Age (y)^a^	57.42 (17.20)
Gender^b^	
Male	87 (58)
Female	63 (42)
Primary ICU admission category^†^	
Medical	70 (46.7)
Surgical	64 (42.7)
Trauma	16 (10.6)
Height (m)^a^	169.17 (8.82)
Weight (kg)^a^	68.57 (9.68)
BMI (kg/m^2^)^a^	22.9 (1.8)
APACHE II score^a^	20.16 (3.82)
SOFA score^a^	8.06 (1.94)
NUTRIC score^b^	
≥5	79 (52.7)
<5	71 (47.3)
Albumin <3 g/dL^b^	91 (60.7)
Prealbumin <15 mg/dL^b^	53 (35.3)

^a^Mean (standard deviation), ^b^ number (%). APACHE II: acute physiology and chronic health evaluation, BMI: body mass index, ICU: intensive care unit, kg: kilograms, m: meters, NUTRIC score: The NUTrition Risk in the Critically ill score, SOFA: Sequential Organ Failure Assessment

According to the results shown in Table 2 enteral nutrition was used for 42.7% of all patients, parenteral nutrition for 28%, and enteral with parenteral nutrition in 29.3%. Moreover, the mean time to begin enteral feeding was 31.4 hours after ICU admission. It should also be mentioned that in 28.6% of our samples enteral feeding was initiated more than 48 hours after ICU admission, and it started later in surgical compared with medical and trauma patients (*P* < 0.001).

**Table 2:** Nutritional related values and outcomes of study patients at the end of study

**Variables**	**Total (N = 150)**
Follow up duration (day)^a^	8.64 (1.50)
Type of Nutrition^b^
Enteral	64 (42.67%)
Parenteral	42 (28%)
Enteral with parenteral	44 (29.33%)
Time to initiate EN from ICU admission (h)^a^	31.40 (40.02)
Prescribed nutrition^a^	
Energy (kcal/kg/d)	17.72 (9.70)
Protein (g/kg/d)	0.99 (0.38)
Delivered nutrition^a^	
Energy (kcal/kg/d)	11.02 (8.17)
Protein (g/kg/d)	0.64 (0.36)
Adequacy of received nutrition^b^	
Calories^c^	
<80%	123 (82.0%)
80-120%	25 (16.7%)
>120%	2 (1.3%)
Protein^c^	
<80%	127 (84.7%)
80-120%	23 (15.3%)
>120%	-
Lipid^d^	
<30%	55 (36.7%)
30-40%	84 (56%)
>40%	11 (7.3%)
Carbohydrate^e^	
<60%	43 (28.7%)
60-70%	62 (41.3%)
>70%	45 (30%)
Prealbumin <15 mg/dL^b^	126 (84%)
Hospital/ICU mortality rate^b^	56 (37.3%)/47 (31.3%)
Duration of hospital/ICU stay^a^	12.14 (7.55)/10.30 (7.55)

^a^Mean (standard deviation); ^b^ number (%); ^c^ Received amounts divided by the estimated daily requirements; ^d^Percent of daily calories provided by lipids; ^e^ Percent of daily calories provided by carbohydrates. EN: enteral nutrition, ICU: intensive care unit.

From the mean of 2053 kcal that theoretically required, 1207 kcal were prescribed. The mean of under-prescription was significantly higher in those received only parenteral nutrition (82% versus 15% and 35% in enteral and combination of enteral and parenteral nutrition, respectively [*P* < 0.001]). In addition, 61.85% and 63.9% of prescribed calories and proteins could be delivered to patients, respectively. In this manner, gastrointestinal intolerance (62%) stopping and restarting enteral feeding because of the diagnostic procedure or mechanical problem (33%), and medical errors (5%) were considered the reasons for these differences.

Administered calories and proteins in 16.7% and 15.3% of patients meet 80%-120% of patients’ daily target requirements, respectively. On the other hand, the optimal amount of lipid and carbohydrates were delivered in 56% and 38.7% of patients, respectively. More information related to underfeeding and overfeeding with respect to energy and protein intakes was described in Table 2. In addition, one sample *t* test showed that the provided amounts of calories and proteins were significantly lower than optimal values (*P* < 0.001). In the case of micronutrients, only 8.5% of patients received vitamins or trace elements. Finally, malnutrition (prealbumin <15 mg/dL) developed in 84% of patients at the end of the patients’ follow-up.

All variables were analyzed to find factors affecting underfeeding and malnutrition by the univariate analysis. Accordingly, a category of medical and surgical patients and those with baseline NUTRIC score ≥5 were associated with underfeeding, and malnutrition was significantly more developed among patients with NUTRIC score ≥5 and surgical patients. More related data and statistically significant variables of multivariate regression can be seen in Table 3.

**Table 3:** Variables associated with underfeeding and malnutrition of ICU patients during the first 10 days

**Variables**	**Univariate regression**		**Multivariate regression**		
	***P*** **value**	**OR**	***P*** **value**	**OR**	**95% CI**
Underfeeding	
Medical patients	<0.001	2.500	0.006	2.614	1.321-5.170
Surgical patients	<0.001	20.330	<0.001	13.714	6.298-70.044
NUTRIC score >5	<0.001	3.158	0.843	0.927	0.440-1.956
Malnutrition
Enteral with parenteral nutrition	0.019	2.143	0.139	2.358	0.756-7.357
Surgical patients	0.003	2.200	0.176	2.224	0.699-7.083
Calorie underfeeding	0.842	0.923	0.355	1.658	0.568-4.839
NUTRIC score ≤ 5	0.145	0.717	<0.001	0.298	0.151-0.586

CI: confidence interval, ICU: intensive care unit, NUTRIC score: The NUTrition Risk in the Critically ill score, OR: odds ratio.

The mortality rate and duration of stay in ICU and hospital can be found in Table 2. According to these results, duration of ICU and hospital stay was not significantly influenced by the nutritional status of the patient. However, underfeeding contributed significantly to more mortality rate both in ICU and hospital (95.7% versus 4.3%, *P* = 0.005 and 94.6% versus 5.4%, *P* = 0.003, respectively).

Hospital malnutrition is a well-identified problem of medical attitude, described more than 20 years ago.^5^ Previous studies reported up to 80% malnutrition among ICU patients in Iran, which were independently related to poorer clinical outcomes in this population.^20^ Various types of institutions and heterogeneous definition for malnutrition are the most reasons which explain a wide range of reported malnutrition prevalence among ICU patients.

Our findings also confirmed this nutritional inadequacy in the critically ill patients. Consequently, more than half of our population was nutritionally at high risk upon admission, and malnutrition was developed in more than 80% of study sample at the end of 10 days. On the other word, unfortunately, majority of nutritionally high-risk patients failed to receive adequate calories and subsequently developed malnutrition. Additionally, more than 80% of our study population failed to meet optimal energy and protein targets. This astonishing protein-energy undernutrition in our study served to precipitate malnutrition. This nutritional decline during the ICU stay is also frequently observed in previous studies, which demonstrated that patients received up to 64% of their required daily energy.^21,22^

The percentage of energy intake divided by energy requirement (determined by physician order, indirect calorimetry, or calculated by the Harris-Benedict equation) was applied for evaluating nutritional adequacy in different studies.^8,13,14,16^ Receiving 80%-120% of energy or proteins target was defined as optimal feeding, which is also mentioned in recent studies.^13,23^ There are some reports that showed that Harris-Benedict equation may overestimate energy expenditure. So, this may explain the low rate of receiving optimal target of energy in our study. Indeed, indirect calorimetry is the reference method to assess resting energy expenditure. Although some discrepancies reported between results of Harris-Benedict equation and indirect calorimetry, the role of indirect calorimetry was limited in clinical studies due to requiring costly equipment and technical skills.^14,24^ Therefore, Harris-Benedict equation is one of the best alternatives in the absence of indirect calorimetry.^25^

In accordance with the previously conducted study, approximately two-thirds of differences in caloric requirement and delivered was explained by under-prescription, and only the remained one third was related to under-delivery.^13^ This low caloric prescription rate was also mentioned in previous studies and necessitates more attention of medical staff to nutritional issues of ICU patients, appropriately using nutritional support algorithm, and referring patients to a clinical pharmacist or ICU dietitian, if needed.^14,26^

This under-prescription was reported from 0%-40% in different studies based on the rout of nutrition administration.^14^ About half of the desired calories were prescribed in this research. However, this is only 18% among patients with only parenteral nutrition. This reflects the inadequate interest of physicians to appropriately feed critically ill patients when enteral nutrition is not feasible, and accentuate the paramount importance of nutritional care as basic support as well as hemodynamic and respiratory care in these patients.

Regarding the time of starting enteral nutrition, in contrast to the previously conducted study, enteral nutrition was initiated less than 48 hours in most patients.^27^ However, these results are echoed in the multicenter international study which showed that enteral feedings were started at the mean of 38.8 hours after ICU admission.^13^

Since not all ICU patients were at risk of underfeeding, more consideration required to identify avoidable reasons that hinder the appropriate feeding.^16^ Related studies mostly focus on nutritional care and describe the distribution of malnutrition and underfeeding from ICU admission until discharge, but did not rigorously assess potentially associated factors.^13,27,28^

We found that surgical patients compared to medical and trauma patients were significantly at risk to develop underfeeding after multivariate regression analysis. This may be related to the gastrointestinal intolerance of these patients after surgical procedures. Later time to start enteral feeding and inefficient parenteral nutrition among surgical patients may be the reasons potentially putting them at risk for underfeeding. Although it is necessary to discriminate high-risk from low-risk patients for delivering feeding based on ASPEN guideline, but unfortunately optimal calories were not provided for nutritionally high-risk patients in our study. This trend could be found in NUTRIC score variable in a univariate analysis which showed that patients who have high malnutrition risk (NUTRIC score ≥5) received lower calories. However, it did not remain significant after multivariate analysis.

There is little in the literature regarding factors affecting caloric intake; however, the recent study indicated that BMI ≥30 kg/m^2^, traumatic brain injury, and gastrointestinal tract injuries are significantly associated with a smaller increase of the caloric intake over time.^16^

On the other hand, an important variable that seemed to be associated with a greater chance of malnutrition was NUTRIC score ≥5 in our analysis. This finding confirms previous results that showed that NUTRIC score is a valid method to identify patients who are at nutritional risk in ICU and may develop malnutrition during the ICU stay.^29^ Arguably, definition of nutritional status in our study limits these results.

However, there is not any unified definition for screening and diagnosing patients with malnutrition, but it is suggested that serum proteins such as prealbumin should be used only in complement to a thorough physical examination in order to determine a patient’s nutritional status.^12^ A recent study declared that serum prealbumin level trends did not correlate with the adequacy of nutrition delivery.^11^ Although our results revealed that energy underfeeding is also associated with malnutrition at the end of the follow-up in univariate model, but this trend was not statistically significant when other variables entered in the multivariate model.

Different studies revealed that detrimental outcomes were independently associated with a nutritional decline during the ICU stay.^30^ Our results also discovered that underfeeding contributed significantly to clinical outcomes such as more ICU and hospital mortality rates. Although patients who are malnourished are prone to these medical complications, they were not statistically significant in our analysis.

Considering the nature of the observational study, no intervention or attempt was conducted to standardize nutritional care in this research. The strength of this study was the prospective design with at least 7-day follow-up and the assessment of feeding pattern using standard definition. In addition, we also introduced variables associated with caloric underfeeding and malnutrition. However, certain limitations also existed in this study. The first was related to the use of prealbumin level for screening and diagnosing patients with malnutrition at the end of the study. However, there is a lack of a unified method to assess malnutrition, and furthermore, prealbumin also has been widely used to evaluate a patient’s nutritional status, but its use has encountered the error in the inflammatory state such as critical condition. Notwithstanding this weakness, NUTRIC score is still able to predict malnutrition defined according to prealbumin level in our study. The second limitation was due to the observational design of the study. Interventional instead of observational studies should be designed to confirm and clarify the relationship between reported risk factors and nutritional values. An additional limitation would be related to not including IL-6 in NUTRIC score calculation and using the modified version of NUTRIC score. Moreover, it should be mentioned that estimated height and dry weight were used for required calculation in our study.

## Conclusion

Different studies showed that, despite the high prevalence of malnutrition on ICU admission, appropriate nutritional care was prescribed to few patients. Malnutrition has not developed in all critically ill patients, and identification of factors associated with malnutrition and inadequate energy intake is essential to improve nutritional support. Therefore, this study offers insights into which ICU patients are at risk for iatrogenic underfeeding and malnutrition. Greater attention to the risk factors such as NUTRIC score as a nutritional risk assessment tool and surgical patients may promote the delivery of calories and proteins in patients who are at risk for underfeeding. Moreover, adequate training of ICU medical staff and multidisciplinary approach regarding nutritional care must be applied to achieve adequate nutritional support in critically ill patients.

## Ethical Issues

This study was in accordance with the ethical standards of the responsible committee on human experimentation and with the Helsinki Declaration of 1975, as revised in 2008. The ethics committee of Isfahan university of medical sciences approved this study. The study was approved by the Isfahan University of Medical Sciences’ ethics board, and patients’ data were kept confidential.

## Conflict of Interest

None to be declared.

## Acknowledgments

We would like to thank staff members of ICU Alzahra hospital for their cooperation (general support). It also should be declared that this study was funded by Isfahan University of Medical Sciences.

## References

[1] Ridley E, Gantner D, Pellegrino V (2015). Nutrition therapy in critically ill patients- a review of current evidence for clinicians. Clin Nutr.

[2] Wray CJ, Mammen JM, Hasselgren PO (2002). Catabolic response to stress and potential benefits of nutrition support. Nutrition.

[3] Lew CCH, Yandell R, Fraser RJL, Chua AP, Chong MFF, Miller M (2017). Association between malnutrition and clinical outcomes in the intensive care unit: a systematic review. JPEN J Parenter Enteral Nutr.

[4] Mogensen KM, Horkan CM, Purtle SW, Moromizato T, Rawn JD, Robinson MK (2017). Malnutrition, critical illness survivors, and postdischarge outcomes: a cohort study. JPEN J Parenter Enteral Nutr.

[5] Havens JM, Columbus AB, Seshadri AJ, Olufajo OA, Mogensen KM, Rawn JD (2018). Malnutrition at intensive care unit admission predicts mortality in emergency general surgery patients. JPEN J Parenter Enteral Nutr.

[6] Heyland DK, Dhaliwal R, Jiang X, Day AG (2011). Identifying critically ill patients who benefit the most from nutrition therapy: the development and initial validation of a novel risk assessment tool. Crit Care.

[7] Kondrup J (2014). Nutritional-risk scoring systems in the intensive care unit. Curr Opin Clin Nutr Metab Care.

[8] Higgins PA, Daly BJ, Lipson AR, Guo SE (2006). Assessing nutritional status in chronically critically ill adult patients. Am J Crit Care.

[9] Arabi YM, Aldawood AS, Al-Dorzi HM, Tamim HM, Haddad SH, Jones G (2017). Permissive Underfeeding or Standard Enteral Feeding in High- and Low-Nutritional-Risk Critically Ill Adults Post Hoc Analysis of the PermiT Trial. Am J Respir Crit Care Med.

[10] Tempel Z, Grandhi R, Maserati M, Panczykowski D, Ochoa J, Russavage J (2015). Prealbumin as a serum biomarker of impaired perioperative nutritional status and risk for surgical site infection after spine surgery. J Neurol Surg A Cent Eur Neurosurg.

[11] Yeh DD, Johnson E, Harrison T, Kaafarani HMA, Lee J, Fagenholz P (2018). Serum Levels of Albumin and Prealbumin Do Not Correlate With Nutrient Delivery in Surgical Intensive Care Unit Patients. Nutr Clin Pract.

[12] Bharadwaj S, Ginoya S, Tandon P, Gohel TD, Guirguis J, Vallabh H (2016). Malnutrition: laboratory markers vs nutritional assessment. Gastroenterol Rep (Oxf).

[13] Heyland DK, Dhaliwal R, Wang M, Day AG (2015). The prevalence of iatrogenic underfeeding in the nutritionally ‘at-risk’ critically ill patient: Results of an international, multicenter, prospective study. Clin Nutr.

[14] De Jonghe B, Appere-De-Vechi C, Fournier M, Tran B, Merrer J, Melchior JC (2001). A prospective survey of nutritional support practices in intensive care unit patients: what is prescribed? What is delivered?. Crit Care Med.

[15] McClave SA, Taylor BE, Martindale RG, Warren MM, Johnson DR, Braunschweig C (2016). Guidelines for the Provision and Assessment of Nutrition Support Therapy in the Adult Critically Ill Patient: Society of Critical Care Medicine (SCCM) and American Society for Parenteral and Enteral Nutrition (ASPEN). JPEN J Parenter Enteral Nutr.

[16] Haltmeier T, Inaba K, Schnuriger B, Siboni S, Benjamin E, Lam L (2018). Factors affecting the caloric and protein intake over time in critically ill trauma patients. J Surg Res.

[17] Hsu PH, Lee CH, Kuo LK, Kung YC, Chen WJ, Tzeng MS (2018). Determination of the energy requirements in mechanically ventilated critically ill elderly patients in different BMI groups using the Harris-Benedict equation. J Formos Med Assoc.

[18] Preiser JC, van Zanten AR, Berger MM, Biolo G, Casaer MP, Doig GS (2015). Metabolic and nutritional support of critically ill patients: consensus and controversies. Crit Care.

[19] Patkova A, Joskova V, Havel E, Kovarik M, Kucharova M, Zadak Z (2017). Energy, protein, carbohydrate, and lipid intakes and their effects on morbidity and mortality in critically ill adult patients: a systematic review. Adv Nutr.

[20] Kimiaei-Asadi H, Tavakolitalab A (2017). The assessment of the malnutrition in traumatic ICU patients in Iran. Electron Physician.

[21] Elpern EH, Stutz L, Peterson S, Gurka DP, Skipper A (2004). Outcomes associated with enteral tube feedings in a medical intensive care unit. Am J Crit Care.

[22] Hejazi N, Mazloom Z, Zand F, Rezaianzadeh A, Amini A (2016). Nutritional assessment in critically ill patients. Iran J Med Sci.

[23] Nicolo M, Heyland DK, Chittams J, Sammarco T, Compher C (2016). Clinical outcomes related to protein delivery in a critically ill population: a multicenter, multinational observation study. JPEN J Parenter Enteral Nutr.

[24] Japur CC, Penaforte FR, Chiarello PG, Monteiro JP, Vieira MN, Basile-Filho A (2009). Harris-Benedict equation for critically ill patients: are there differences with indirect calorimetry?. J Crit Care.

[25] Walker RN, Heuberger RA (2009). Predictive equations for energy needs for the critically ill. Respir Care.

[26] Woien H, Bjork IT (2006). Nutrition of the critically ill patient and effects of implementing a nutritional support algorithm in ICU. J Clin Nurs.

[27] Stewart ML, Biddle M, Thomas T (2017). Evaluation of current feeding practices in the critically ill: A retrospective chart review. Intensive Crit Care Nurs.

[28] Dobson K, Scott A (2007). Review of ICU nutrition support practices: implementing the nurse-led enteral feeding algorithm. Nurs Crit Care.

[29] Mendes R, Policarpo S, Fortuna P, Alves M, Virella D, Heyland DK (2017). Nutritional risk assessment and cultural validation of the modified NUTRIC score in critically ill patients-A multicenter prospective cohort study. J Crit Care.

[30] Elke G, Wang M, Weiler N, Day AG, Heyland DK (2014). Close to recommended caloric and protein intake by enteral nutrition is associated with better clinical outcome of critically ill septic patients: secondary analysis of a large international nutrition database. Crit Care.

